# Electrospun Bioresorbable Membrane Eluting Chlorhexidine for Dental Implants

**DOI:** 10.3390/polym12010066

**Published:** 2020-01-02

**Authors:** Pierre Pouponneau, Ophélie Perrey, Céline Brunon, Carol Grossiord, Nicolas Courtois, Vincent Salles, Antoine Alves

**Affiliations:** 1 Statice, 25000 Besançon, France; o.perrey@statice.com; 2 Science et Surface, 69130 Écully, France; c.brunon@serma.com (C.B.); c.grossiord@serma.com (C.G.); 3 Anthogyr, 74700 Sallanches, France; n.courtois@anthogyr.com; 4 Univ Lyon, Université Claude Bernard Lyon1, Laboratoire des Multimatériaux et Interfaces, UMR CNRS 5615, F-69622 Villeurbanne, France; vincent.salles@univ-lyon1.fr; 5 Namsa, 38670 Chasse-sur-Rhône, France; aalves@namsa.com

**Keywords:** electrospinning, bioresorbable polymers, dental membrane, drug delivery, peri-implantitis

## Abstract

To prevent the uncontrolled development of a pathogenic biofilm around a dental implant, an antimicrobial drug-release electrospun membrane, set up between the implant and the gingival tissue, was developed by taking several technical, industrial and regulatory specifications into account. The membrane formulation is made of a blend of poly(l-lactic–*co*–gycolic acid) (PLGA, 85:15) and poly(l-lactic acide–*co*–ɛ-caprolactone) (PLC, 70:30) copolymers with chlorhexidine diacetate (CHX) complexed with β-cyclodextrin (CD). The amount of residual solvent, the mechanical properties and the drug release kinetics were tuned by the copolymers’ ratio, between 30% and 100% of PLC, and a CHX loading up to 20% *w*/*w*. The membranes were sterilized by γ-irradiation without significant property changes. The fiber′s diameter was between 600 nm and 3 µm, depending on the membrane composition and the electrospinning parameters. CHX was released in vitro over 10 days and the bacterial inhibitory concentration, 80 µg·mL^−1^, was reached within eight days. The optimal membrane, PGLA/PLC/CHX-CD (60%/40%/4%), exhibited a breaking strain of 50%, allowing its safe handling. This membrane and a membrane without CHX-CD were implanted subcutaneous in a rat model. The cell penetration remained low. The next step will be to increase the porosity of the membrane to improve the dynamic cell penetration and tissue remodeling.

## 1. Introduction

Implant dentistry is one of the best-performing surgical treatments, with excellent long-term clinical success data. Very good success rates—as high as 97%—after 10 years are regularly reported in significant cohorts [1]. These results demonstrate the very good risk/benefit balance of a treatment, which is very efficient in restoring patients′ quality of life. However, although most long-term studies are performed by high-skilled teams with properly taught patients, peri-implant infection remains a major cause of complications [2,3]. Peri-implant infection, referred to as peri-implantitis, is linked to the uncontrolled development of a pathogenic biofilm and may result in severe bone loss around the implant as a result of local tissue inflammation.

As the use of implant treatment increases worldwide, with approximately 25 million implants placed annually, and considering that the prevalence of this pathology is high [4], fighting peri-implantitis and improving its treatment must be considered a key objective to maintaining implant performance level. The origin of the acute inflammatory process causing tissue resorption resides in the proliferation of pathogenic bacteria in the non-cleanable transgingival zone. The disease evolution from peri-implant mucositis to peri-implantitis is associated with a progressive loss of contact between the connective tissue and the implant surface, enabling the biofilm to move down into the peri-implant pocket.

Peri-implantitis treatments generally encompass surgical debridement and decontamination, in order to heal the inflammatory lesion. Implantoplasty, consisting of the mechanical smoothening of the contaminated implant surface, recently appeared as a radical but rather efficient method [5]. However, this method appears to be a one-shot option, as it considerably changes the implant topography, therefore modifying the implant’s mechanical properties [6].

Moreover, to prevent recidivism of this inflammatory pocket, securing a soft tissue-regenerative approach, in combination with the initial recommended antibacterial treatment with chlorhexidine (CHX) as a firm adhesion between the gingiva and the implant, is highly desirable to prevent bacteria moving down the endosseous implant zone [7]. The smooth transgingival zone is the theater of the so-called “race for the surface” [8], where soft-tissue adhesion competes with biofilm formation. Hence, it appears favorable to guide soft tissue growth towards the implant surface while maintaining an unfavorable environment for bacteria. Such adhesion could be achieved by a membrane setup between the implant and the gingiva tissue. Moreover, the membrane could release an antiseptic agent to prevent early bacterial infection during the tissue integration step.

Electrospinning is an adequate process to manufacture this kind of membrane because this process allows the generation of a material made of fibers which can be tuned to improve cell interaction and colonization and the release of a drug. Electrospun membranes for dental applications have been previously reported [9,10]. Several studies focus on the treatment of parodontitis by the development of membranes for guided tissue/bone regeneration [9]. To the best of our knowledge, few studies investigate the development of an electrospun coating or membrane around an implant to prevent prei-implantitis [3]. Only the inhibition of the biofilm formation, induced by a peri-implantitis-associated pathogen, with the release of tetracycline hydrochloride (TCH) loading into gelatine-poly(l-lactic acid (PLLA) and poly(ɛ-caprolactone) (PCL) electrospun fibers, has been investigated [3].

In this work, we have investigated the design and manufacturing of an electrospun bioresorbable membrane eluting an antiseptic agent according to several technical, industrial and regulatory specifications: (i) absence of residual solvent, (ii) sterilization with an industrial process, (iii) elution of the drug over several days, (iv) mechanical properties appropriate for the handling and suture of a dental implant and (v) electrospinning stability for manufacturing scale-up. These specifications, drawn from clinical need and the standards for medical device market approval, significantly increase the technical challenges of developing such a membrane.

To manage all these specifications, we have investigated different bioresorbable copolymers such as poly(l-lactic–*co*–gycolic acid) (PLGA) and poly(l-lactic acide–*co*–ɛ-caprolactone) (PLC). PLGA is a well-known polymer for drug release [9]. We report here the design of this membrane—a blend of PLGA and PLC with CHX complexed by 2-Hydroxylpropyl)-β-cyclodextrine (CD)—the properties of several electrospun membranes according to their formulation and their electrospinning parameters, the selection of the sterilization method, the in vitro CHX release study, the mechanical properties and the first in vivo study, where local tissue effects were investigated.

## 2. Materials and Methods 

### 2.1. Materials

PLGA8523 (l-lactide/glycolide comonomer ratio: 85/15 mol%, 2.4 dL·g^−1^) and PLC7015 (l-lactide/caprolactone comonomer ratio: 70/30 mol%, 1.5 dL·g^−1^) were purchased from Corbion (Netherlands). Chlorhexidine diacetate (CHX), 2-Hydroxylpropyl-β-cyclodextrine (CD) and phosphate buffer saline (PBS) powder were purchased from VWR (France). 1,1,1,3,3,3-Hexafluoro-2-propanol (HFIP) was purchased from Apollo Scientific (UK).

### 2.2. CHX Complexation with CD

CHX (1.5 g·L^−1^) and CD (molar ratio: 1:1) were stirred in pure water at room temperature 24 h prior to freeze drying for five days.

#### 2.2.1. Membrane Electrospinning

PLC and PLGA (100:0; 70:30; 60:40; 50:50; 40:60; 30:70 (% *w*/*w* polymer weight)), CHX (0% to 20% *w*/*w* polymer weight) and CD (CHX:CD 1/1 or 1/4 molar ratio) were blended and stirred in HFIP (100 mg·mL^−1^) overnight. The solution was electrospun on a rotating target (100 rpm) with an industrial system (EC-CLI, IME, Netherlands) at 30 °C with a humidity rate of 20%. Depending on the formulation, the distance between the emitter and the target was between 14 and 18 cm, the flow rate was 3 mL·h^−1^, the emitter voltage was between 10 and 16 kV and the target voltage was −4 kV. After electrospinning, the patch (270 mm × 60 mm) was dried at 40 °C in a heating cabinet from between 4 and 10 days to remove residual solvent. Samples were cut in the patch.

#### 2.2.2. Membrane Sterilization

Samples were packaged in Tyvek^®^ pouches (DuPont, France). Gamma-irradiation sterilization at 10–15 kGy was used (Steris, France). Ethylene oxide (EtO) sterilization (3 h) was done at a low temperature (30–40 °C) (Sterlab, France). EtO residue, measured by gas chromatography, was below 0.1 mg per sample (Sterlab).

### 2.3. Scanning Electron Microscopy (SEM)–Energy Dispersive X-ray Spectroscopy (EDS)

The surface morphology and chemical composition were evaluated using a scanning electron microscope (Quanta 250-FEG (FEI), Hillsboro, OR) operating at an accelerating voltage of 1 kV and equipped with an SDD Brucker detector. Detection limits of EDS analyses depend on the elements and matrix and were below 0.1 at %. In this work, the detection of the Fluor element, used to track solvent residues in the membrane, was below 0.1 at % and corresponded to less than 0.1 wt %, measured by thermogravimetric analysis. Fiber diameters (at least 30 measures) were measured on four areas of the sample with ImageJ software (National Institues of Heath (NIH), 1.48 VVersion, US).

### 2.4. X-ray Photoelectron Spectroscopy (XPS)

Measurements were done using a PHI Quantera SXM instrument (Physical Electronics, Chanhassen, MN) equipped with a 180° hemispherical electron energy analyzer and a monochromatized Al Kα (1486.6 eV) source operated at 15 kV and 4 mA. The analysis spot had a diameter of 200 μm and the detection angle relative to the substrate surface was 45°. Standard deviations were calculated from measurements performed on two different areas. Data were analyzed using the Multipak software (Version 9.8.0.19, Physical Electronics GmbH, Ismaning, Germany). The depth probe of XPS analysis was between 5 and 10 nm.

### 2.5. Fourier-Transform Infrared Spectroscopy (FTIR)

FTIR analyses were performed using a Spotlight 400 microspectrometer (Perkin–Elmer, Waltham, MA). FTIR mapping was performed in Attenuated Total Refection (ATR) mode using the ATR-imaging accessory with a germanium crystal. The scanned area was 400 × 400 µm² (lateral resolution of 1.56 µm). The Mercury-Cadmium-Telluride (MCT) detector (Perkin–Elmer, Waltham, MA) covered the range 720–4000 cm^−1^ with a spectral resolution of 8 cm^−1^. Principal Component Analysis (PCA) calculation was applied to raw FTIR images using Spectrum IMAGE software (Perkin–Elmer).

### 2.6. Differential Scanning Calorimetry (DSC)

Experiments were carried out in a DSC 1 (Mettler Toledo, Columbus, OH). Each sample was cut and introduced (weight = 5 ± 0.5 mg) in an aluminum crucible (40 µL). Then, it was cooled down to −40 °C and subsequently heated up to measure the thermal signal between −20 and +200 °C under nitrogen (N2), with a ramp of 10 °C·min^−1^.

### 2.7. In Vitro Drug Release Study

Samples (n = 6, Ø12 mm) were immerged under sink conditions in PBS (15 mL, 1×, pH = 7.4) at 37 °C under moderate stirring. The release of the CHX in PBS was monitored with a UV-spectrometer (VWR, France) at λ = 254 nm. Each day, after the measurement, PBS was changed. The same results were achieved with the immersion of one membrane in PBS (3 mL, n = 3) under sink conditions.

### 2.8. Mechanical Characterization

The membrane thickness was measured with a laser system (Keyence, France). Samples (n = 3, dog-bone tensile specimens (60 mm × 10 mm)) were tested at 3.3 mm·min^−1^ with a tensile system (Multitest1-X, Mecmesin limited, Horsham, UK) updated with an acquisition system (Andilog, Vitrolles, France). Data were analyzed with a Wilcoxon signed-rank test (*p* < 0.05).

### 2.9. In Vivo Test

#### 2.9.1. Animal Protocol

Two female rats (*Rattus norvegicus,* Charles River Laboratories France) weighing 325 ± 60 g (mean ± SD), were involved in the present study. The rats were individually housed in stainless steel suspended cages under laboratory conditions (humidity and temperature recorded daily). Humidity was maintained higher than 30% of relative humidity and temperature was maintained between 20 and 24 °C. The artificial light cycle was controlled using an automatic timer (12 h of light, 12 h of dark). Standard rodent feed and water were provided ad libitum. The implantation was carried out by accommodating the membranes in subcutaneous pockets parallel to the vertebral column. The study was divided into two groups of sites: a group with a membrane devoid of CHX and a group with a membrane loaded with 4% of CHX-CD. For each formulation, the membranes (Ø 12, 253 ± 31 µm, 5 ± 0.3 mg, n = 6) were implanted for 20 days under standard aseptic conditions. Both membrane groups (n = 3 per group) were implanted in one animal.

The protocol of the present study was consistent with the requirements of the European legislation for the protection of animals used for scientific purposes (Directive 2010/63/EU). It was approved by the local NAMSA Ethical Committee, as NAMSA is an accredited facility registered at the French Department of Agriculture for animal housing, care and investigations.

At termination, histopathological analysis was conducted after macroscopic observations.

#### 2.9.2. Histologic Preparation

After complete fixation in 10% neutral buffered formalin (NBF) for 24 to 48 h, a total of 12 implanted sites, together with two implanted T = 0 sites (one from each membrane formulation at termination) were dehydrated in alcohol solutions of increasing concentration, cleared in xylene and embedded in paraffin. One central section per site was cut using a microtome (4–7 µm thickness). This section was stained with safranin–hematoxylin–eosin (SHE). 

#### 2.9.3. Histopathologic Evaluation

Qualitative and semi-quantitative histopathologic evaluation of the local tissue effects and integration of the membranes was adapted to the standard and conducted (ISO 10993—Part 6). Histopathology grading score was defined as follows: 0 = None, 1 = Slight, 2 = Moderate, 3 = Marked, 4 = Severe/Complete.

## 3. Results and Discussion

### 3.1. Membrane Formulation Design 

For this project, electrospinning was done with a polymer–solvent solution, because this will avoid the early degradation of the bioresorbable polymers and the antiseptic agent due to processing with heat [11]. The residual amount of solvent in the membrane has to be very low to avoid cytotoxic effects [12]. Furthermore, solvents, which can be carcinogens and neurotoxins, have to be avoided for the safety of the manufacturing operators, the dental surgeon and the patient.

The sterilization has to be investigated in the first developmental steps; it can significantly change the scaffold properties [13,14]. This critical process can decrease the mechanical properties, which are required to ensure the handling and the setup of the membrane. Furthermore, sterilization can induce chemical modifications, leading to significant changes in the polymer biodegradation rate and drug activity. In this work, gamma irradiation and ethylene oxide sterilization, two processes classified with a high inactivation level on microorganisms [13] and widely used to sterilize medical devices, are investigated.

Chlorhexidine is an antiseptic agent used in dental treatments such as endodontic therapy [15]. This agent is effective against both gram-positive and gram-negative microbes. It is supposed that its guanidium groups can bind to the negatively charged bacteria cell walls, leading to bacteriostatic and bactericidal activity. It was reported that a high local drug concentration is required to eliminate bacteria initially, which has to be followed by a sustained long-term drug release to prevent secondary infections. Without this drug release profile, more drug-resistant biofilms could be formed [16]. Hence, our membrane should release the antiseptic agent over several days to prevent infection around the implant [17].

The membrane will be sutured around the implant. Hence, the mechanical properties should be adequate to avoid membrane tearing: low breaking stress and strain values could be an issue.

The size of the membrane will be 20 mm × 5 mm × 0.2 mm. From a manufacturing point of view, this membrane will be cut in a larger patch (at least 180 mm × 250 mm × 0.2 mm). Hence, the electrospinning of the patch could take several hours. The process has to be stable to avoid local defects′ formation.

The membrane formulation was designed to meet all these specifications. To reach our aim, different bioresorbable copolymers were electrospun (Table 1). Regarding polymer choice, we have mainly focused on the following technical and regulatory specifications: (i) absence of residual solvent, (ii) elution of the drug over several days and (iii) mechanical properties appropriate for membrane handling.

The electrospun PLGA membranes exhibit solvent residues despite a drying step at 40 °C over a period of several days or under vacuum (Table 1, sample A). As HFIP was used as an electrospinning solvent, the residual amounts found in the membrane should be very low [12]. HFIP was chosen because it is considered a good solvent for electrospinning, especially for long periods [18]. In this project, the membranes will be cut in a large patch (270 mm × 180 mm), requiring several hours of electrospinning. Moreover, the electrospun PLC membranes exhibit no remaining residual solvent after drying (Table 1, sample B).

Solvent retention is a critical property, due to the toxicity of the solvent used during electrospinning [19]. It was reported that electrospun PGA fibers retained a higher amount of HFIP than that measured with electrospun PLC fibers [19]. HFIP is progressively removed from PLC fibers until complete evaporation, which is not the case for PGA electrospun fibers, where remaining solvent was still detected after 14 days [19]. Moreover, PCL has no significant interaction with HFIP (2,3), whereas PLLA has an affinity [20]. It was suggested that solvent retention can be linked to polymer glass transition temperature (*T*_g_): polymers with *T*_g_ above room temperature could retain solvent because solvent diffusion out of the fibers during the electrospinning is limited by the decrease in polymer chain mobility. These studies confirm our findings, as electrospun PLGA fibers retain HFIP despite the evaporation step.

Furthermore, electrospun PLGA membranes are more rigid than PLC membranes. Therefore, it could be challenging to suture them around the implant. To confirm the choice of PLC for the manufacturing of the membrane, we have investigated CHX release (Section 3.4). The drug release profile was characterized by a strong burst of release, near to half of the loading, followed by a very low elution. The elution was stopped after two days. Consequently, to optimize the mechanical properties, the drug release profile and to minimize the residual amount of solvent, we have blended PLGA with PLC. The electrospun membranes based on these formulations were free of residual solvent (Table 1, samples C, F, G and H).

The chlorhexidine diacetate is poorly soluble in physiological solutions, due to the chloride concentration, leading to its precipitation [15,21,22]. Consequently, the minimum effective concentrations for antimicrobial activity cannot be reached. Hence, to ensure its release from the electrospun membrane, CHX was complexed with CD, a cyclic oligosaccharide made of glucopyranoside units exhibiting a cup-like structure. It was reported that the hydroxyl groups on the outer surface of CD make it hydrophilic, whereas the inner cavity is hydrophobic, allowing the trapping of guest molecules and formation of host–guest inclusion complexes. Hence, CHX forms a host–guest inclusion complex with CD [23]. Furthermore, it was reported that this complex exhibits an inhibitory effect on dental bacteria [24,25] and can be released from a PLGA matrix [21]. Moreover, CD can be electrospun with aliphatic polyester [26,27].

**Figure 1:**
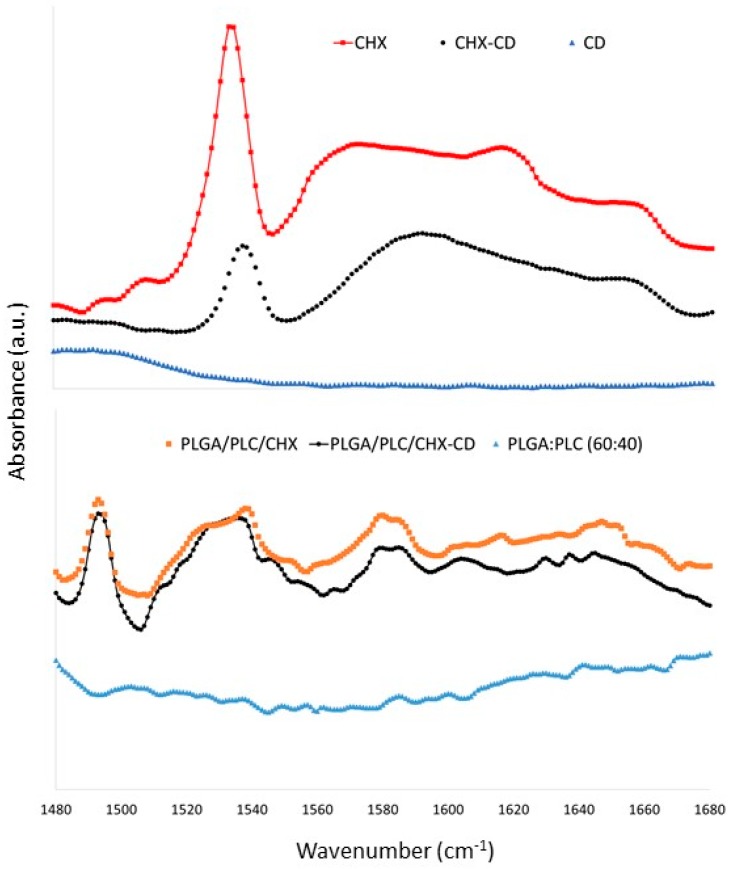
Fourier-transform infrared spectroscopy (FTIR) spectrum of the CHX, CD, the complex CHX-CD, the membrane PLGA/PLC: (60:40), the membrane PLGA/PLC/CHX (60:40:4) and the membrane PLGA/PLC/CHX-CD (60:40:4).

We have confirmed the presence of CHX-CD complex in the membrane by FTIR and DSC analysis (Figure 1 and Figure S1 in Supplementary Materials). Compared to the spectra of CHX and CD, variations in the intensity and the position of the bands of C=C groups in CHX aromatic rings were detected on CHX-CD spectrum: a decrease in the intensity CHX band at 1550 and 1510 cm^−1^ and a shift in the CHX band from 1614 to 1604 cm^−1^ (Figure 1). These changes, which have been previously reported [23,28], could be induced by the potential breakdown of C=C bonds or the variation in the chemical environment of the rings, induced by a chemical interaction between CHX and CD molecules. Moreover, the presence of CHX in the fibers was confirmed on the DSC curves by the two endothermic peaks of around 50 and 155 °C (Figure S1 in Supplementary Materials). Those peaks were present in the DSC curves of the samples containing CHX and CD, and a new endothermic signal was detected around 70 °C which could be attributed to the presence of CD. This result is consistent with a previous study on the same CD-CHX complex [25]. According to this last study, the only difference that could be found between the samples containing a complex CHX-CD and those without, was a new endothermic peak that appeared at 239 °C. The slight decrease in the curve just before 200 °C is in line with the phenomenon of complexation (Figure S1 in Supplementary Materials).

**Figure 2:**
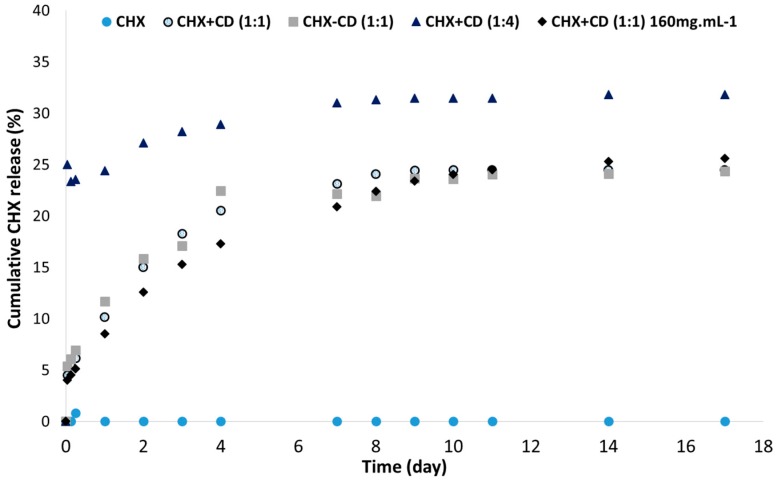
Cumulative CHX release (%) through time according to the complexation of CHX with CD and polymers′ concentrations, as measured by UV spectrometry. The formulation used was PLGA:PLC (60/40) with CHX (4%). CHX + CD means CHX and CD were added together during the polymers′ solubilization. The lyophilized CHX-CD complex powder was added during the polymers′ solubilization.

To confirm the design of the membrane formulation, we have investigated the effect of CD on CHX release in vitro (Figure 2). Without CD, no release of CHX was detected. We have investigated two strategies to prepare the electrospinning solution: (i) the lyophilized CHX-CD complex powder, added to the polymers in HFIP or (ii) CHX and CD, added to the polymers in HFIP. With both strategies, CHX was released from the membrane with the same profile. The complexation of CHX can occur in HFIP, despite the presence of polymers.

We then investigated the impact of CD in excess. The membrane exhibits a strong burst release of CHX. However, solvent residues were detected (Table 1, Sample N). 

The increase in the polymers′ concentration had no significant effect on the drug release profile. However, the fiber diameter significantly increased and residual solvent was detected (Table 1, sample M).

The design of the membrane formulation was validated. Then, different electrospun membranes were characterized.

### 3.2. Membrane Characterizations

**Figure 3:**
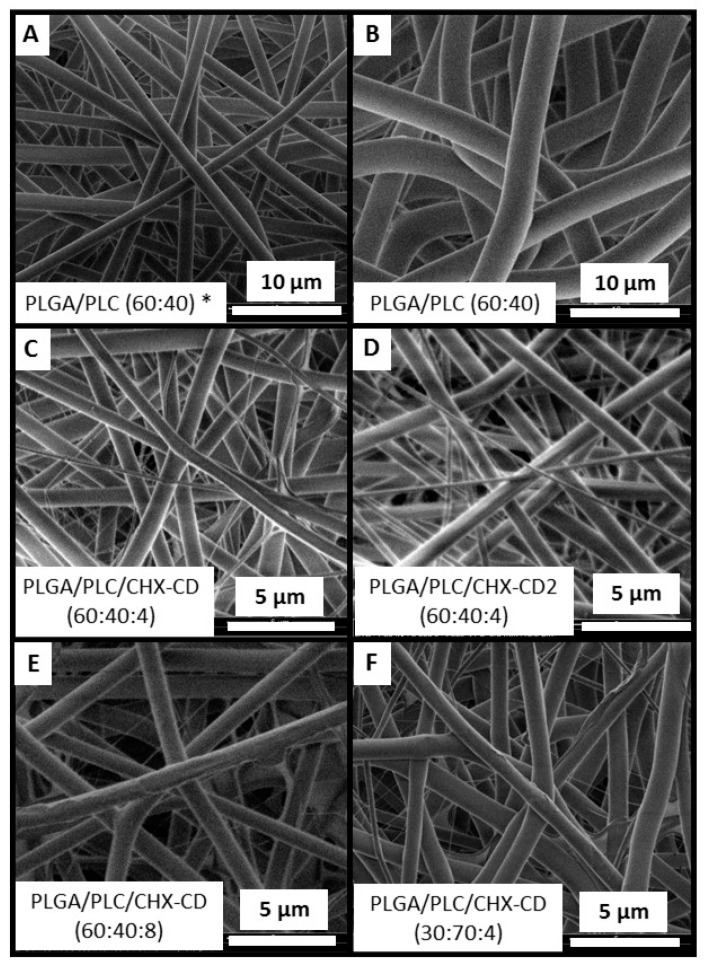
SEM images of fibers according to their formulations. Mean fiber diameter: (**A**) 1458 ± 174 nm; (**B**) 2996 ± 189 nm; (**C**) 655 ± 366 nm; (**D**) 642 ± 270 nm; (**E**) 824 ± 362 nm; (**F**) 746 ± 297 nm. In (**A**), fibers were electrospun with a 0.4 mm capillary. In (**D**), the lyophilized CHX-CD complex powder was added into the polymers solution.

We have investigated the fibers′ morphologies according to their composition. Without CHX, fiber diameter was around 1.5 µm (Figure 3, Table 1 ref C). It can be increased by up to 3 µm by changing the capillary diameter from 0.4 to 0.6 mm (Table 1, sample E). The fibers were free of beads.

When CHX and CD were added, the fiber diameter decreased significantly by around 0.7 µm, with a broad distribution. Small fibers (200 ≤ Ø ≤ 400 nm) were created. The decrease in fiber diameter could be attributed to the change in the solution′s conductivity due to the presence of CHX, as already observed with another protein and polymer system [29]. Moreover, the electrospinning of a formulation with only CD did not significantly change the fiber’s diameter (Table 1, sample D).

The use of lyophilized CHX-CD complex powder in the preparation of the electrospinning solution had no significant change on the fiber′s morphology, compared to those achieved with the electrospinning solution made by adding PLGA, PLC, CHX and CD (Figure 3C,D, Table 1, sample H,L).

The increase in the CHX content did not significantly change the fiber’s morphology. The mean diameter increased slightly with CHX content. Fibers with a diameter below 400 nm are still imaged (Figure 3E). The minor increase in fiber diameter with the drug content could be due to the increase in the solution viscosity induced by the increase in CHX and CD [3].

Furthermore, when the content of CHX was increased above 8% (Table 1, sample J,K), the presence of the Fluor element, attributed to HFIP, was detected. When only CD was added to the formulation, no significant change in the fiber morphology was detected, but residual solvent was measured in the membrane (Table 1, sample D). When the ratio CHX:CD was increased from 1:1 to 1:4, the fiber diameter distribution was not significantly changed, but residual solvent was detected in the membrane (Table 1, sample N). We could assume that CD may interact with HFIP.

When the polymer concentration was increased up to 160 mg·mL^−1^, the mean fiber diameter increased up to 2 µm (Table 1, sample M). The fiber diameter distribution was reduced, and no bimodal distribution was detected. However, residual solvent was detected in the membrane.

XPS analyses were done on two areas on the surface of the samples (Table 2 and Table 3). Within the sensitivity of the technique (0.1 to 0.5 at %), no other elements were detected in the spectra. The atomic concentrations, obtained from high-resolution spectra, have confirmed the absence of Fluor on the surface of the membranes (Table 2).

The C1s photopeak, obtained from high resolution spectra, shows several contributions: at 285 eV, corresponding to C–C, C–H bonds, at 286.7 eV, corresponding to C–O and/or C–N bonds, at 287.8 eV, corresponding to C=O and/or O–C–O and/or O–C–N bonds, and at 288.6 eV, corresponding to O–C=O and/or O–C=N and/or N–C=O and/or N–C=N bonds (Table 3). These analyses show an increase in the nitrogen content (in organic form) and in chlorine (mainly in organic form), which are attributed to CHX (increase in N/Cl ratio). The strong presence of C–O and C–N bonds at the surface of PLGA/PLC/CD and PLGA/PLC/CHX-CD has confirmed the detection of CD. Hence, the CHX-CD element was detected on the fiber surface.

### 3.3. Selection of the Sterilization Method 

**Figure 4:**
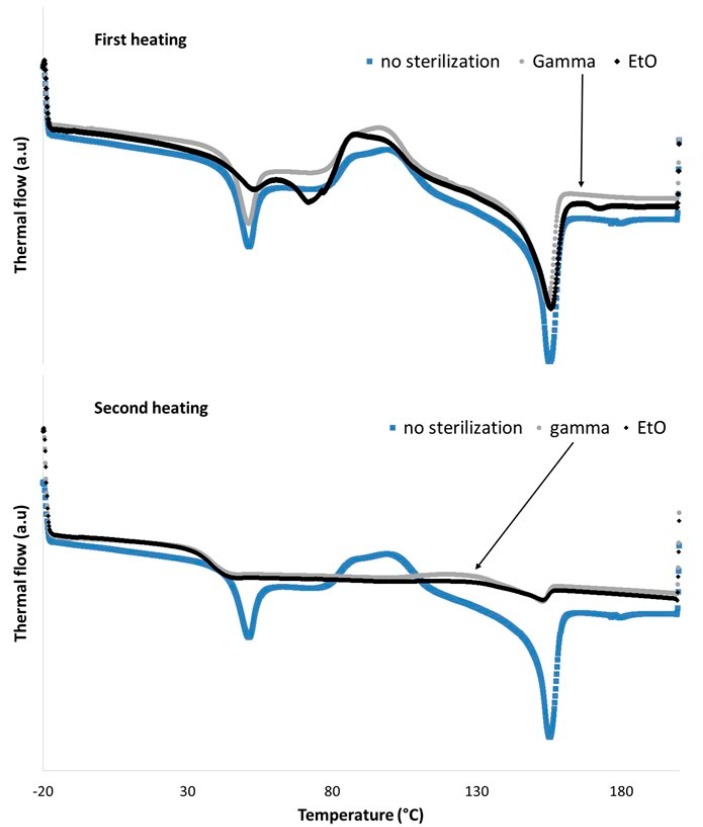
DSC curves of the PLGA/PLC/CHX (60:40:4) membranes before and after sterilization with gamma irradiation or EtO treatment. The two graphics show the results after the first heating cycle (**top**) and the second heating step (**bottom**).

The DSC curves obtained in this study (Figure 4) are in good agreement with the ones obtained with PLC copolymer powders [30] and pellets made of PLGA 85:15 [31]. The first heating of the as-spun membrane revealed the first endothermic phenomenon, corresponding to the glass transition, with an onset at 44 °C for samples without sterilization and after ɣ-irradiation. For the membrane sterilized with EtO, onset was at 40 °C and the endothermic phenomenon was divided into two peaks. This difference was not visible after the second heating step with an onset of about 32 °C for the three samples. Moreover, the peak, centered at 50 °C, observed during the first heating, has disappeared. This is attributed to the polymer chain relaxation. This phenomenon was previously reported and explained by comparing the DSC curves of pellets and the electrospun filaments of PCL [32].

At higher temperatures, the fibers tended to crystallize from 100 °C (exothermic phenomenon) before melting at around 142 °C (onset). The thermal flow, which is required to melt the membranes without any sterilization, reached 6.22 J·g^−1^, whereas the samples sterilized with gamma irradiation and with EtO reached 2.96 and 1.47 J·g^−1^, respectively. These last values were calculated from the areas of the corresponding peaks, obtained during the second heating step. From these results, we can conclude that the sterilization step induced a non-negligible effect on the polymer chains. Although both techniques had an effect, the impact of the EtO treatment was more significant than the gamma irradiation, taking into account the enthalpies mentioned above. Furthermore, it was reported that, even after irradiation with a dose of 15 kGy, electrospun PCL fibers exhibit a higher hydrophilicity than ones before irradiation, due to the partial cleavage of ester groups to form hydroxyl (–OH) and carboxyl (–COOH) functional groups [33]. On the same kind of polymer, a partial crosslinking can also occur but seems to be limited even after irradiation at a higher dose (25 kGy) [34]. The same dose used on PLGA fibers induces a decrease of about 50% of molecular weight [35] and the impact is even worse with higher doses [36]. PLC contains about 30% of PCL and 70% of PLLA. Moreover, ethylene oxide sterilization could induce a risk of toxicity due to residual ethylene oxide confined in the polymer [14].

**Figure 5:**
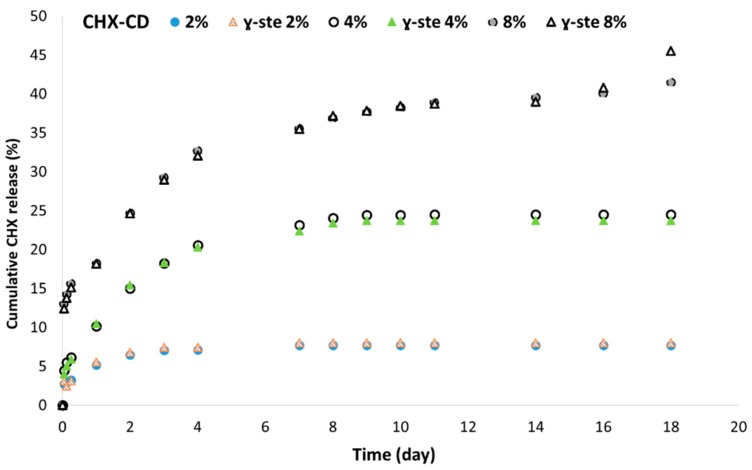
Cumulative CHX release according to the CHX loading with the formulation PLGA/PLC/CHX-CD (60:40) before and after sterilization by γ-irradiation, measured by UV spectrometry.

Furthermore, the chemical structure of the CHX was not modified after ɣ-irradiation sterilization (Figure S2 in Supplementary Materials). No significant change was seen in the drug release profile after sterilization (Figure 5). The effects of the sterilization observed in this work are in good agreement with the literature [13,14]. Hence, selecting gamma irradiation (10–15 kGy) as the sterilization method for the rest of the study was a good compromise.

### 3.4. In Vitro CHX Release

We have investigated CHX release according to the polymers′ ratio and CHX-CD content. We have compared the daily CHX concentration release to the minimum inhibitory concentration.

**Figure 6:**
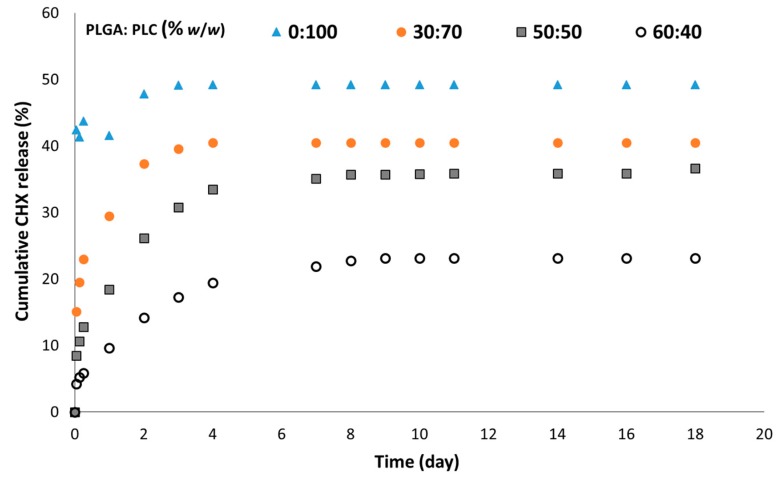
Cumulative CHX release according to the PLGA/PLC ratio. CHX-CD loading was 4%, measured by UV spectrometry.

For the same CHX loading, when the content of PLC increased by more than 50% (*w*/*w*), the amount of drug released increased (Figure 6). However, the drug release profile was characterized by a strong burst release, and elution was stopped after two days. Below this value, the CHX was released over several days (Figure 6).

**Figure 7:**
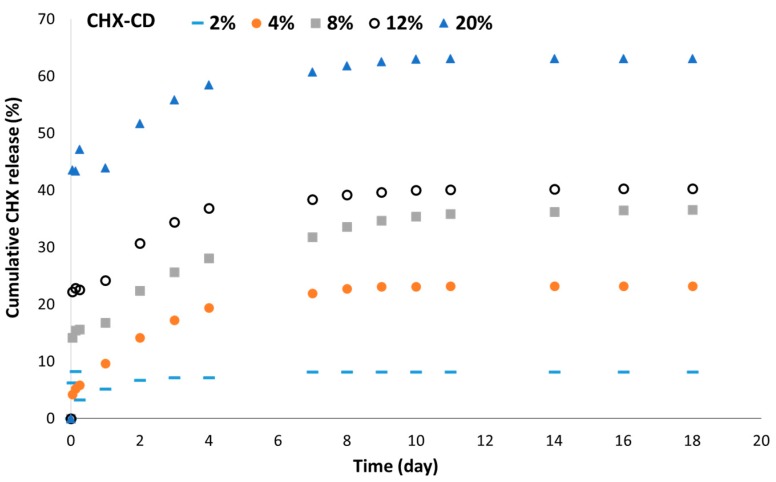
Cumulative CHX release according to the CHX-CD loading with the formulation PLGA/PLC (60:40), measured by UV spectrometry.

The amount of initial drug release was related to the CHX content (Figure 7). The burst release increased with CHX loading. This effect of the drug content on the burst release was previously reported in a different electrospun membrane [37]. Moreover, for a loading above 2%, the drug was released over 8–10 days. With a CHX loading at or below 2%, the amount of drug released remained very low (Figure 7).

With all formulations, the remaining amount of CHX was not released. This result was previously observed with electrospun CHX-PLLA fibers [16] or PLGA matrix [21]. It was beyond the scope of this work to investigate the mechanism which controls drug release. We compared our results with the literature to explain our drug release kinetic. As reported in [33], we guess that the polymer–drug interaction might be responsible for the steady state of CHX release observed after several days of elution [38]. When the drug content increases, the amount of the drug not linked to the polymer could increase, and would thus be released during the first days. Hence, it could be assumed that a portion of the drug, which can increase with the drug content, is desorbed and diffused out through the physiological solution-filled pores. The remaining portion of the CHX is probably trapped in the fiber crystalline areas, which the physiological solution cannot access until polymer degradation [39]. The drug release kinetic achieved with these membranes could be divided into three stages: (i) the immediate drug dissolution on the fiber surface, (ii) the drug desorption and diffusion through the pores and (iii) the release with the polymer degradation. With our formulation, it is expected that degradation will only start after several months.

**Figure 8:**
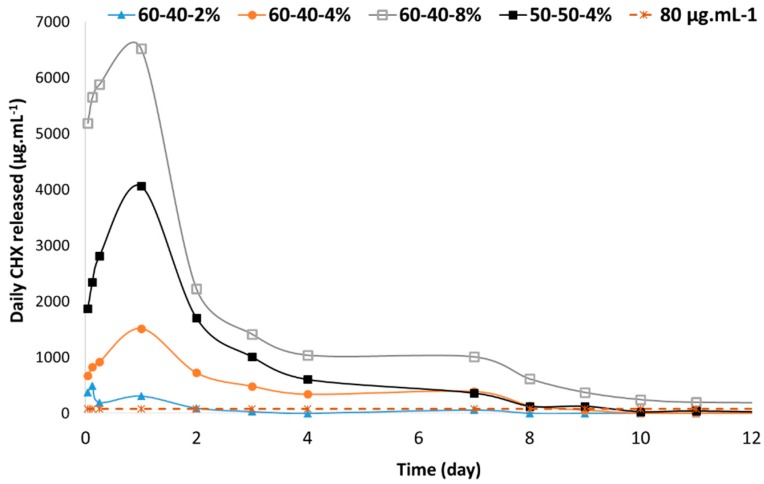
Daily CHX concentration, measured by UV spectrometry, released by a membrane (115 mm^3^) according to the formulation (polymer ratio and CHX loading).

It was reported that the minimum inhibitory concentration of chlorhexidine for different anaerobic oral bacteria was between 3 and 80 µg·mL^−1^ [25,40]. We have compared these values with the daily CHX concentration released by one membrane (Figure 8). Hence, to ensure the release of the inhibitory concentration over several days, the membrane should be loaded at a minimum of 4%. By changing the polymer ratio, the daily CHX concentration in the first few days can be significantly increased. This result could be interesting in the case of a serious infection. Hence, the membrane formulation, loaded at a minimum of 4%, could induce an antibacterial effect over the course of several days and the dose released during the first day can be tuned according to the polymer ratio. The antibacterial effect could be limited after 10 days. Moreover, this in vitro study confirms the release of CHX. However, it should be noted that the peri-implant space is a complex environment characterized by a low volume, with body fluids and healthy or inflamed tissues which can modify the pharmacokinetic. An in vivo study could provide more information on the drug release profile.

### 3.5. Mechanical Properties

**Figure 9:**
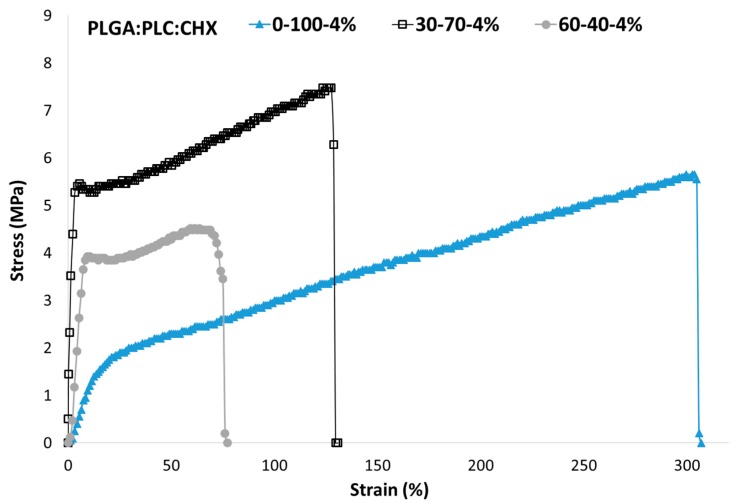
Stress–strain curve according to the ratio between PLGA and PLC copolymers for a CHX loading of 4%.

**Figure 10:**
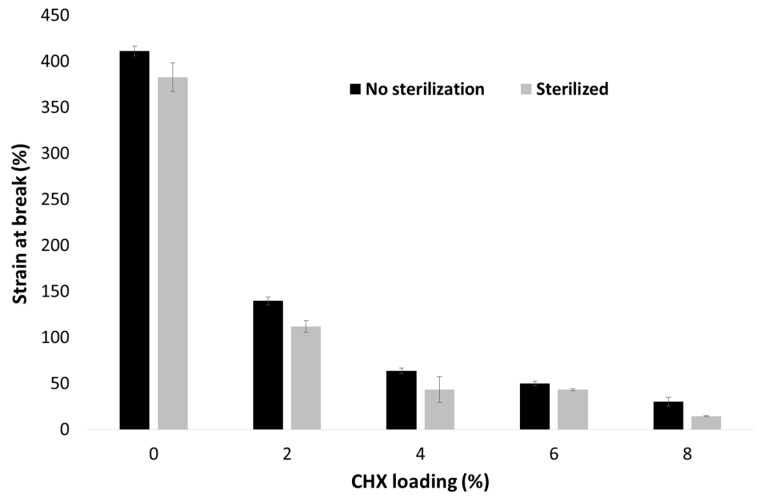
Strain at break according to the loading of CHX before and after sterilization (*p* = 0.118) for PLGA/PLC (60:40) membrane.

The handling of the membrane, depending on its formulation, was investigated with an in vitro tensile test. PLC material is characterized by a breaking strain above 300% [41,42]. The addition of CHX in the membrane has reduced the mechanical properties (Figure 9 and Figure 10). When the content of PLC decreased, the rigidity of the membrane increased, and the breaking stress decreased. With a PLGA:PLC ratio of 60:40, the breaking strain and stress remained above 50% and 4 MPa, respectively, which should be sufficient to ensure the suture of the membrane around the implant.

When the loading of CHX increased from 2% to 8%, the breaking strain of sterilized membranes decreased from 112% to 14% (Figure 10). However, the decrease in the stress at break with the increase in CHX content was moderate (Figure S3 in Supplementary Materials). Hence, with a loading of 8%, the mechanical properties of the membrane could be considered at risk for the suture around the implant. After the γ-irradiation, the strain at break was slightly reduced, but remained acceptable for the membrane handling.

The mechanical results found in this work were in good agreement with the literature [43]. The blend of two polymers provides hybrid mechanical properties. PLGA is known for its stiffness and PCL exhibits a higher strain at break than PLGA due to the ɛ-caprolactone content. Furthermore, it was reported that the increase of CHX content decreases the mechanical properties of PLLA electrospun fibers [16].

### 3.6. In Vivo Study

Based on the in vitro data, we have investigated the local tissue effects induced by one formulation: PLGA/PLC/CHX-CD: 60:40/4%. We chose this CHX content to minimize cell toxicity. Furthermore, it could induce an inhibitory effect on oral bacteria (Figure 8) [16]. The mechanical properties seem appropriate for the membrane handling. As a control, a formulation without CHX was used.

No remarkable events occurred during the in vivo phase. For both types of membrane, after 20 days of sub-cutaneous implantation, no significant dimensional changes were measured, and there were similarly slight signs of cell-mediated degradation.

**Figure 11:**
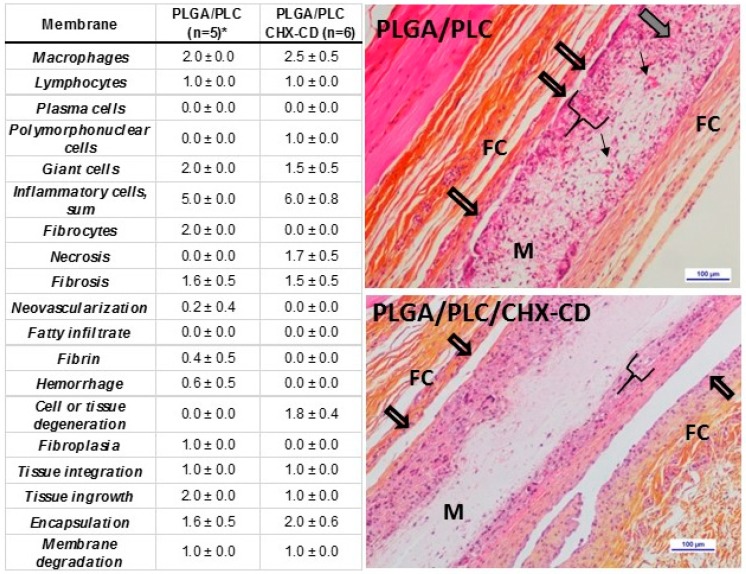
Semi-quantitative histopathologic analysis table (n = number of membranes analyzed, mean ± SD) after 20 days of sub-cutaneous implantation and photomicrographs of the implanted membranes. Legend: allow arrows: no local tissue integration, bracket: extend of cells tissue ingrowth, black arrows: neovessels, grey arrow: active fibroblasts, M: membrane, FC: Fibrous encapsulation. * One sample was damaged during the technical preparation.

With the PLGA/PLC control membrane (Figure 11 and Figure S4 in Supplementary Materials), the inflammatory reaction was characterized by a moderate grade of macrophages and giant cells admixed with a slight number of lymphocytes. No significant signs of necrosis were detected. A moderate grade of cell ingrowth, including a moderate infiltration of fibroblasts secreting a slight amount of collagen fibers, was observed. The membrane was perfused by a few neovessels. Slight to moderate signs of fibrous encapsulation, despite the cell ingrowth, resulted in a slight tissue integration of the control membrane.

With PLGA/PLC membrane loaded with 4% CHX-CD, the inflammatory reaction was slightly higher compared to the one induced by the control membrane. It was characterized by a moderate to marked grade of macrophages, a slight to moderate grade of giant cells admixed with a slight number of polymorphonuclear cells and lymphocytes. A slight to moderate cell necrosis (including coagulative necrosis) and cell degeneration were detected. The cell ingrowth was slight and no fibrocytes or collagen deposit were detected within the membrane. Moderate signs of fibrous encapsulation, despite the cell ingrowth, resulted in a slight tissue integration of the membrane.

We attributed the low cell colonization of the membrane loaded with CHX to its low porosity (higher density of fibers constituted of micro or nanofibers) compared to the control, which was only constituted of microfibers. The cell necrosis observed at the loaded membrane contact was partly related to the absence of blood supply (no neovessels formed within the loaded membrane). On the other hand, a slight toxic effect of the loaded membrane (occurrence of coagulative necrosis) should not be ruled out. Therefore, both the low porosity and the CHX dose might explain the low cell penetration into the loaded membrane when compared to the unloaded membrane.

To improve these preliminary in vivo results, we could try to decrease the fiber density in the membrane loaded with CHX-CD. It was reported that scaffolds with micro or nanofibers with a lower fiber density lead to better cell proliferation and infiltration [44,45]. Several strategies have been reported to achieve loose fibrous structures and large pores to enhance cell filtration [45]. For this project, we will investigate strategies without the removal of sacrificial fibers, because the membrane cannot be wet prior to the sterilization in order to avoid early CHX release and fiber degradation.

## 4. Conclusions

The electrospun dental membrane introduced in this paper was designed and developed to achieve multiple therapeutic purposes. The design of the membrane formulation meets the technical, industrial and regulatory specifications. Several membrane formulations based on bioresorbable copolymers were investigated. The blend of two copolymers allows for the tuning of the mechanical properties and the drug release profile. The antiseptic agent was complexed to ensure its release in physiological solutions. Formulation-related parameters, such as the copolymers ratio, CHX/CD ratio and CHX-CD content, exhibit a direct impact on the properties of the membrane, such as the fiber diameter, residual solvent content, drug release profile and mechanical properties.

A moderate drug loading of around 4% seems appropriate for the mechanical properties required for the membrane handling and the drug release profile. The membrane can be sterilized by industrial γ-irradiation and its manufacturing scale-up could be achieved. The in vivo results showed that cell penetration was low. Given the moderate number of cells involved in the interfacial necrotic process, it is believed that increasing the porosity of the loaded membrane will improve the dynamics of cell penetration, neovascularization, cell renewal and tissue remodeling. It is then hypothesized that the level of necrosis will decrease, lowering the local biological risk and increasing the antibacterial benefits of this membrane. Our next step will be to optimize the fiber diameter and the porosity of the membrane by tuning the electrospinning parameters. Furthermore, we will check the biodegradation rate of the membrane and the inhibition of biofilm formation through time according to the drug release profile.

## Figures and Tables

**Table 1:** Fiber diameter, Fluor concentration and membrane thickness, according to the formulation (% w/w), copolymers with chlorhexidine diacetate (CHX) content (% w/w) and CHX/CD ratio.

Sample	Concentration (mg·mL^−1^)	PLGA (%)	PLC (%)	CHX (%)	Ratio CD	F (at %)	Fiber Ø (nm)	Thickness (µm)
A	75	100	0	0	0	0.85	1877 ± 331	239 ± 20
B	80	0	100	10	0	0	1264 ± 138	217 ± 21
C	100	60	40	0	0	0	1458 ± 174 *	259 ± 23
D	100	60	40	0	1	<0.1	1532 ± 237 *	268 ± 57
E	100	60	40	0	0	0	2996 ± 189	227 ± 12
F	100	30	70	4	1	0	746 ± 297	199 ± 19
G	100	50	50	4	1	0	897 ± 450	285 ± 41
H	100	60	40	4	1	0	655 ± 366	265 ± 30
I	100	60	40	6	1	0	664 ± 320	290 ± 37
J	100	60	40	8	1	<0.1	824 ± 369	351 ± 31
K	100	60	40	20	1	0.75	967 ± 173	398 ± 10
L	100	60	40	4	CD2	0	642 ± 270	234 ± 18
M	160	60	40	4	1	0.4	2283 ± 223	409 ± 39
N	100	60	40	4	4	1.05	889 ± 343	296 ± 26

* A 0.4 mm capillary was used, instead of the 0.6 mm capillary used for the manufacturing of the different membranes. CD2 refers to the use of the lyophilized CHX-CD powder.

**Table 2:** Elemental composition (at %) measured by XPS on the PLGA/PLC (60:40) membrane, PLGA/PLC (60:40) membrane with CD (4%), PLGA/PLC (60:40) membrane with CHX-CD (4%) and CHX, CH, CHX-CD powder.

Sample	C (%)	O (%)	N (%)	Cl (%)	O/C (%)	N/Cl (%)
PLGA/PLC (60:40)	65.1 ± 1.2	34.5 ± 1.3	0.5 ± 1.3	-	0.53 ± 0.03	-
PLGA/PLC/CD (60:40:4)	59.7 ± 0.1	40.1 ± 0.1	0.2 ± 0.1	-	0.67 ± 0.01	-
PLGA/PLC/CHX-CD (60:40:4)	62.0 ± 0.1	36.2 ± 0.4	1.7 ± 0.3	0.2 ± 0.1	0.59 ± 0.01	6.92 ± 0.82
CHX	70.6 ± 0.2	8.9 ± 0.7	17.0 ± 0.6	3.5 ± 0.3	0.13 ± 0.01	4.88 ± 0.21
CD	57.8 ± 0.4	42.2 ± 0.4	-	-	0.73 ± 0.01	-
CHX-CD	56.6 ± 2.2	33.4 ± 1.6	7.9 ± 0.5	2.1 ± 0.1	0.59 ± 0.05	3.73 ± 0.08

**Table 3:** Elemental C1s photopeak synthesis measured by XPS on the PLGA/PLC (60:40) membrane, PLGA/PLC (60:40) membrane with CD (4%), PLGA/PLC (60:40) membrane with CHX-CD (4%) and CHX, CHX, CHX-CD powder.

Sample	C–C/C–H	C–O/C–N	C=O/O–C–O/ O–C–N	O–C=O/O–C=N/ N–C=O/N–C=N
PLGA/PLC (60:40)-pt1	43	25	5	27
PLGA/PLC (60:40)-pt2	43	27	2	28
PLGA/PLC/CD (60:40:4)-pt1	27	45	18	10
PLGA/PLC/CD (60:40:4)-pt2	27	45	18	10
PLGA/PLC/CHX-CD (60:40:4)-pt1	34	35	13	18
PLGA/PLC/CHX-CD (60:40:4)-pt2	30	34	18	18
CHX-pt1	68	20	2	10
CHX-pt2	65	23	2	10
CD-pt 1	14	60	21	5
CD-pt 2	14	65	17	4
CHX-CD-pt 1	22	53	21	4
CHX-CD-pt 2	24	56	17	3

## Supplementary Materials

The following are available online at https://www.mdpi.com/2073-4360/12/1/66/s1, Figure S1: DSC curves of electrospun PLGA/PLC (60:40) membrane without CHX, and with CHX in presence or not of CD; Figure S2: FTIR analysis of CHX before and after ɣ-irradiation sterilization; Figure S3: stress at break according to the loading of CHX before and after sterilization for PLGA/PLC (60:40) membrane; Figure S4: SEM images of the histological samples: 60:40%-PLGA/PLC membrane (top) and PLGA/PLC/CHX-CD (60:40:4) membrane (bottom). The circles identify the cells in the membrane.

## References

[1] Buser D., Janner S.F.M., Wittneben J.-G., Brägger U., Ramseier C.A., Salvi G.E. (2012). 10-year survival and success rates of 511 titanium implants with a sandblasted and acid-etched surface: A retrospective study in 303 partially edentulous patients. Clin. Implant Dent. Relat. Res..

[2] van Velzen F.J.J., Ofec R., Schulten E.A.J.M., Ten Bruggenkate C.M. (2015). 10-year survival rate and the incidence of peri-implant disease of 374 titanium dental implants with a SLA surface: A prospective cohort study in 177 fully and partially edentulous patients. Clin. Oral Implants Res..

[3] Shahi R.G., Albuquerque M.T.P., Münchow E.A., Blanchard S.B., Gregory R.L., Bottino M.C. (2017). Novel bioactive tetracycline-containing electrospun polymer fibers as a potential antibacterial dental implant coating. Odontology.

[4] Salvi G.E., Cosgarea R., Sculean A. (2017). Prevalence and Mechanisms of Peri-implant Diseases. J. Dent. Res..

[5] Romeo E., Lops D., Chiapasco M., Ghisolfi M., Vogel G. (2007). Therapy of peri-implantitis with resective surgery. A 3-year clinical trial on rough screw-shaped oral implants. Part II: Radiographic outcome. Clin. Oral Implants Res..

[6] Sahrmann P., Luso S., Mueller C., Ender A., Attin T., Stawarczyk B., Schmidlin P.R. (2019). Titanium Implant Characteristics After Implantoplasty: An In Vitro Study on Two Different Kinds of Instrumentation. Int. J. Oral Maxillofac. Implants.

[7] Heitz-Mayfield L.J.A., Needleman I., Salvi G.E., Pjetursson B.E. (2014). Consensus statements and clinical recommendations for prevention and management of biologic and technical implant complications. Int. J. Oral Maxillofac. Implants.

[8] Gristina A.G. (1987). Biomaterial-centered infection: Microbial adhesion versus tissue integration. Science.

[9] Zhuang Y., Lin K., Yu H. (2019). Advance of Nano-Composite Electrospun Fibers in Periodontal Regeneration. Front. Chem..

[10] Reise M., Wyrwa R., Müller U., Zylinski M., Völpel A., Schnabelrauch M., Berg A., Jandt K.D., Watts D.C., Sigusch B.W. (2012). Release of metronidazole from electrospun poly(L-lactide-co-D/L-lactide) fibers for local periodontitis treatment. Dent. Mater..

[11] Tijing L., Woo Y.C., Yao M., Ren J., Shon H.K. (2017). Electrospinning for Membrane Fabrication: Strategies and Applications. Reference Module in Chemistry, Molecular Sciences and Chemical Engineering.

[12] Nam J., Huang Y., Agarwal S., Lannutti J. (2008). Materials selection and residual solvent retention in biodegradable electrospun fibers. J. Appl. Polym. Sci..

[13] Dai Z., Ronholm J., Tian Y., Sethi B., Cao X. (2016). Sterilization techniques for biodegradable scaffolds in tissue engineering applications. J. Tissue Eng..

[14] Rediguieri C.F., Sassonia R.C., Dua K., Kikuchi I.S., de Jesus Andreoli Pinto T. (2016). Impact of sterilization methods on electrospun scaffolds for tissue engineering. Eur. Polym. J..

[15] Zeng P., Rao A., Wiedmann T.S., Bowles W. (2009). Solubility properties of chlorhexidine salts. Drug Dev. Ind. Pharm..

[16] Luo D., Zhang X., Shahid S., Cattell M.J., Gould D.J., Sukhorukov G.B. (2016). Electrospun poly(lactic acid) fibers containing novel chlorhexidine particles with sustained antibacterial activity. Biomater. Sci..

[17] Pokrowiecki R. (2018). The paradigm shift for drug delivery systems for oral and maxillofacial implants. Drug Deliv..

[18] Chung S., Ingle N.P., Montero G.A., Kim S.H., King M.W. (2010). Bioresorbable elastomeric vascular tissue engineering scaffolds via melt spinning and electrospinning. Acta Biomater..

[19] D’Amato A.R., Bramson M.T.K., Puhl D.L., Johnson J., Corr D.T., Gilbert R.J. (2018). Solvent retention in electrospun fibers affects scaffold mechanical properties. Electrospinning.

[20] D’Amato A.R., Schaub N.J., Cardenas J.M., Fiumara A.S., Troiano P.M., Fischetti A., Gilbert R.J. (2017). Removal of Retained Electrospinning Solvent Prolongs Drug Release from Electrospun PLLA Fibers. Polym. Guildf.

[21] Yue I.C., Poff J., Cortés M.E., Sinisterra R.D., Faris C.B., Hildgen P., Langer R., Shastri V.P. (2004). A novel polymeric chlorhexidine delivery device for the treatment of periodontal disease. Biomaterials.

[22] Zeng P., Zhang G., Rao A., Bowles W., Wiedmann T.S. (2009). Concentration dependent aggregation properties of chlorhexidine salts. Int. J. Pharm..

[23] Lavoine N., Tabary N., Desloges I., Martel B., Bras J. (2014). Controlled release of chlorhexidine digluconate using β-cyclodextrin and microfibrillated cellulose. Colloids Surf. B Biointerfaces.

[24] Sousa F.B.D., Denadai Â.M.L., Lula I.S., Nascimento C.S., Neto N.S.G.F., Lima A.C., Almeida W.B.D., Sinisterra R.D. (2008). Supramolecular Self-Assembly of Cyclodextrin and Higher Water Soluble Guest: Thermodynamics and Topological Studies. J. Am. Chem. Soc..

[25] Cortés M.E., Sinisterra R.D., Avila-Campos M.J., Tortamano N., Rocha R.G. (2001). The Chlorhexidine: Beta;-Cyclodextrin Inclusion Compound: Preparation, Characterization and Microbiological Evaluation. J. Incl. Phenom..

[26] Narayanan G., Shen J., Boy R., Gupta B.S., Tonelli A.E. (2018). Aliphatic Polyester Nanofibers Functionalized with Cyclodextrins and Cyclodextrin-Guest Inclusion Complexes. Polymers.

[27] Topuz F., Uyar T. (2019). Electrospinning of Cyclodextrin Functional Nanofibers for Drug Delivery Applications. Pharmaceutics.

[28] Pupe C.G., Villardi M., Rodrigues C.R., Rocha H.V.A., Maia L.C., de Sousa V.P., Cabral L.M. (2011). Preparation and evaluation of antimicrobial activity of nanosystems for the control of oral pathogens Streptococcus mutans and Candida albicans. Int. J. Nanomed..

[29] Jiang H., Fang D., Hsiao B.S., Chu B., Chen W. (2004). Optimization and characterization of dextran membranes prepared by electrospinning. Biomacromolecules.

[30] Fernández J., Etxeberria A., Sarasua J.-R. (2012). Synthesis, structure and properties of poly(L-lactide-co-ε-caprolactone) statistical copolymers. J. Mech. Behav. Biomed. Mater..

[31] Davison L., Themistou E., Buchanan F., Cunningham E. (2018). Low temperature gamma sterilization of a bioresorbable polymer, PLGA. Radiat. Phys. Chem..

[32] Rychter M., Baranowska-Korczyc A., Milanowski B., Jarek M., Maciejewska B.M., Coy E.L., Lulek J. (2018). Cilostazol-Loaded Poly(ε-Caprolactone) Electrospun Drug Delivery System for Cardiovascular Applications. Pharm. Res..

[33] Augustine R., Saha A., Jayachandran V.P., Thomas S., Kalarikkal N. (2015). Dose-Dependent Effects of Gamma Irradiation on the Materials Properties and Cell Proliferation of Electrospun Polycaprolactone Tissue Engineering Scaffolds. Int. J. Polym. Mater. Polym. Biomater..

[34] Cottam E., Hukins D.W.L., Lee K., Hewitt C., Jenkins M.J. (2009). Effect of sterilisation by gamma irradiation on the ability of polycaprolactone (PCL) to act as a scaffold material. Med. Eng. Phys..

[35] Holy C.E., Cheng C., Davies J.E., Shoichet M.S. (2000). Optimizing the sterilization of PLGA scaffolds for use in tissue engineering. Biomaterials.

[36] Selim M., Bullock A.J., Blackwood K.A., Chapple C.R., MacNeil S. (2011). Developing biodegradable scaffolds for tissue engineering of the urethra. BJU Int..

[37] Chou S.-F., Carson D., Woodrow K.A. (2015). Current strategies for sustaining drug release from electrospun nanofibers. J. Control Release.

[38] Priyadarshini B.M., Mitali K., Lu T.B., Handral H.K., Dubey N., Fawzy A.S. (2017). PLGA nanoparticles as chlorhexidine-delivery carrier to resin-dentin adhesive interface. Dent. Mater..

[39] Natu M.V., de Sousa H.C., Gil M.H. (2010). Effects of drug solubility, state and loading on controlled release in bicomponent electrospun fibers. Int. J. Pharm..

[40] do Amorim C.V.G., Aun C.E., Mayer M.P.A. (2004). Susceptibility of some oral microorganisms to chlorhexidine and paramonochlorophenol. Braz. Oral Res..

[41] Niu Z., Wang X., Meng X., Guo X., Jiang Y., Xu Y., Li Q., Shen C. (2019). Controllable fiber orientation and nonlinear elasticity of electrospun nanofibrous small diameter tubular scaffolds for vascular tissue engineering. Biomed. Mater..

[42] Jundziłł A., Pokrywczyńska M., Adamowicz J., Kowalczyk T., Nowacki M., Bodnar M., Marszałek A., Frontczak-Baniewicz M., Mikułowski G., Kloskowski T. (2017). Vascularization Potential of Electrospun Poly(L-Lactide-co-Caprolactone) Scaffold: The Impact for Tissue Engineering. Med. Sci. Monit..

[43] Chou S.-F., Woodrow K.A. (2017). Relationships between mechanical properties and drug release from electrospun fibers of PCL and PLGA blends. J. Mech. Behav. Biomed. Mater..

[44] Soliman S., Sant S., Nichol J.W., Khabiry M., Traversa E., Khademhosseini A. (2011). Controlling the porosity of fibrous scaffolds by modulating the fiber diameter and packing density. J. Biomed. Mater. Res. A.

[45] Wu J., Hong Y. (2016). Enhancing cell infiltration of electrospun fibrous scaffolds in tissue regeneration. Bioact. Mater..

