# Supplementary material for: Electrospun Bioresorbable Membrane Eluting Chlorhexidine for Dental Implants
Source: Polymers (Basel). 2020 Jan 2;12(1):66. doi: 10.3390/polym12010066 (PMC7023585; doi:10.3390/polym12010066)
Supplement: Supplementary file 1 [file polymers-12-00066-s001.docx]

**Supporting Information**


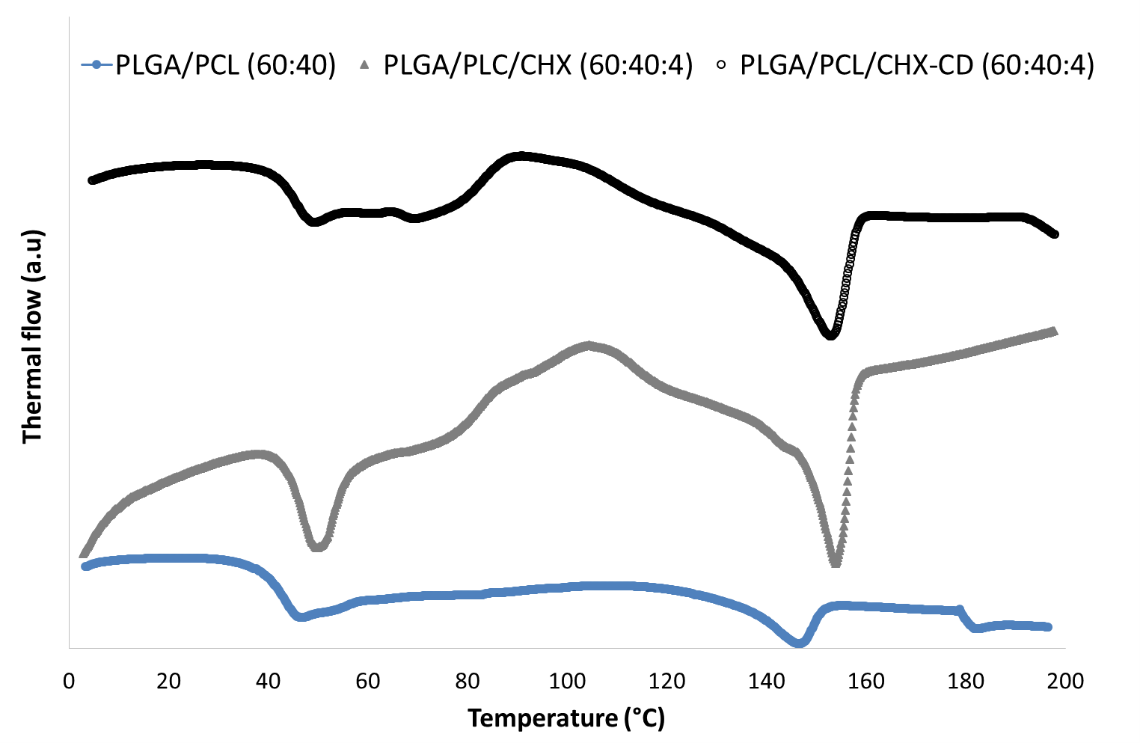


**Figure S1:** DSC curves of electrospun PLGA/PLC (60:40) membrane without CHX, and with CHX in presence or not of CD.


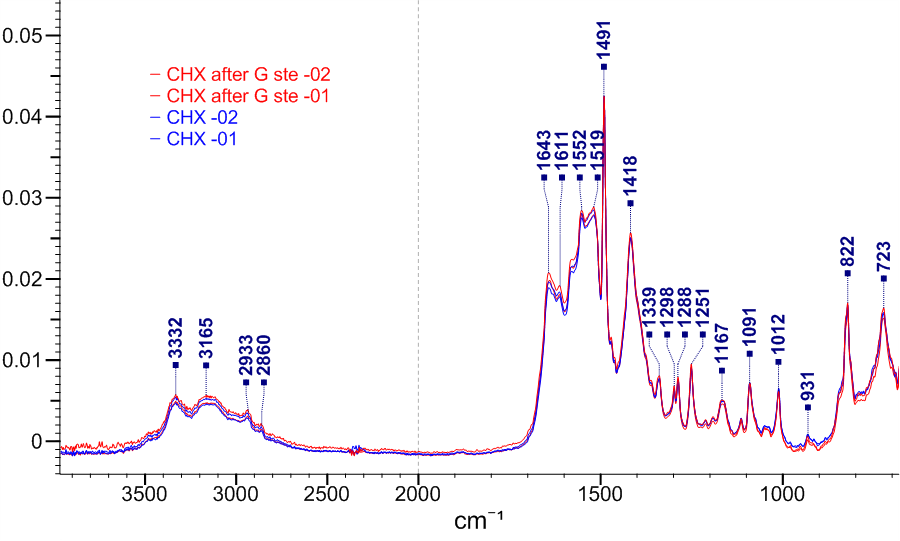


**Figure S2:** FTIR analysis of CHX before and after ɣ-irradiation sterilization


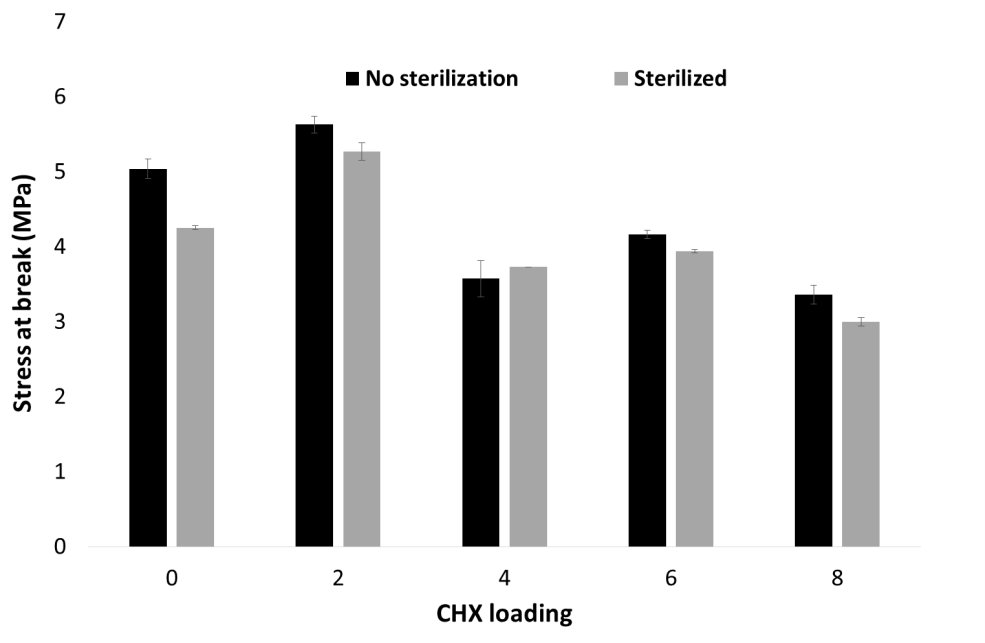


**Figure S3: S**tress at break according to the loading of CHX before and after sterilization for PLGA/PLC (60:40) membrane**.**


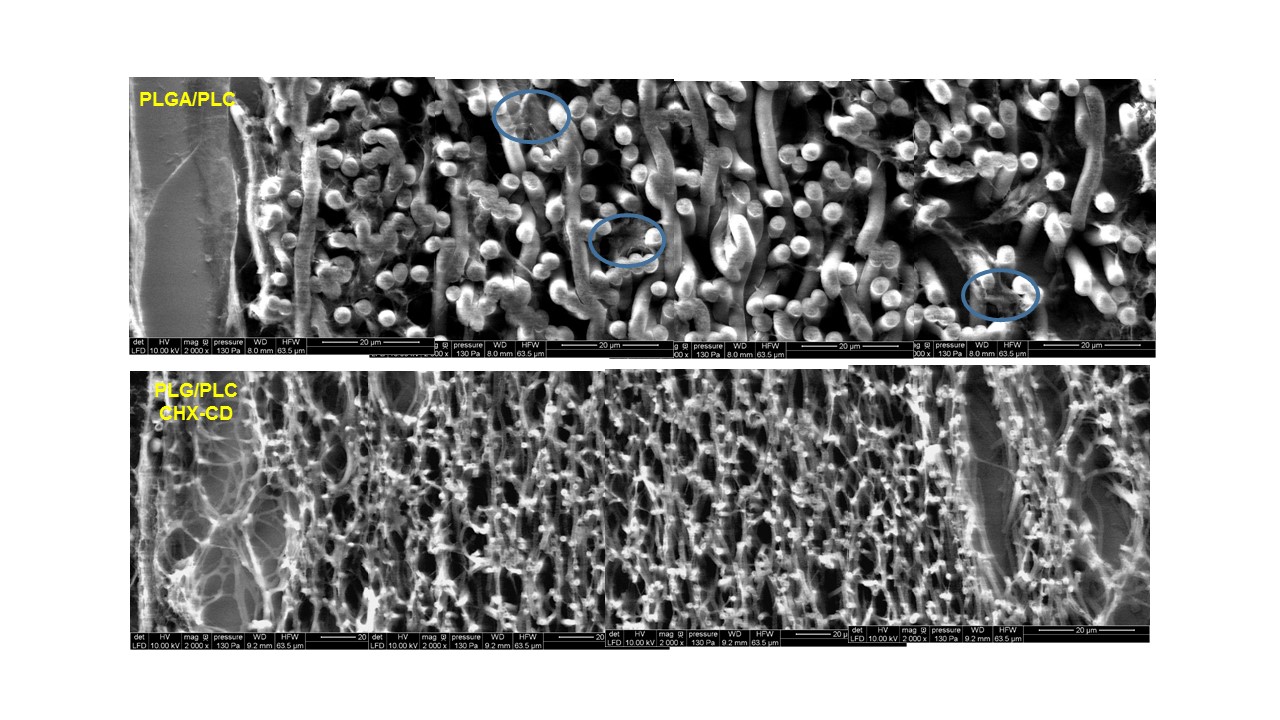


**Figure S4:** SEM images of the histological samples: 60:40%-PLGA/PLC membrane (top) and PLGA/PLC/CHX-CD (60:40:4) membrane (bottom). The circles identify cells in the membrane.
